# Environmental assessment of interventions to restrain the impact of industrial pollution using a quasi-experimental design: limitations of the interventions and recommendations for public health policy

**DOI:** 10.1186/s12889-021-11832-3

**Published:** 2021-10-14

**Authors:** Emilio A. L. Gianicolo, Marco Cervino, Antonello Russo, Susanne Singer, Maria Blettner, Cristina Mangia

**Affiliations:** 1 Division of Epidemiology and Health Services Research, Working Group for the Evaluation of Political Intervention, Institute of Medical Biostatistics, Epidemiology and Informatics (IMBEI), University Medical Center of the Johannes Gutenberg University Mainz, Obere Zahlbacher Str. 69, 55131 Mainz, Germany; 2 Istitute of Clinical Physiology, National Research Council, Lecce, Italy; 3 Istitute of Atmospheric Sciences and Climate, National Research Council, Bologna, Italy; 4 Istitute of Atmospheric Sciences and Climate, National Research Council, Lecce, Italy

**Keywords:** Evaluation of interventions, Air quality, Steel industry, Taranto (southern Italy), Interrupted time series

## Abstract

**Background:**

In an industrial area, the asymmetry between the weights of the economic interests compared to the public-health needs can determine which interests are represented in decision-making processes. This might lead to partial interventions, whose impacts are not always evaluated. This study focuses on two interventions implemented in Taranto, Italy, a city hosting one of the largest steel plants in Europe. The first intervention deals with measures industrial plants must implement by law to reduce emissions during so called “wind days” in order to reduce PM_10_ and benzo [a] pyrene concentrations. The second one is a warning to the population with recommendations to aerate indoor spaces from 12 pm to 6 pm, when pollutant concentrations are believed to be lower.

**Methods:**

To analyse the impact of the first intervention, we analysed monthly PM_10_ data in the period 2009–2016 from two monitoring stations and conducted an interrupted-time-series analysis. Coefficients of time-based covariates are estimated in the regression model. To minimise potential confounding, monthly concentrations of PM_10_ in a neighbourhood 13 km away from the steel plant were used as a control series. To evaluate the second intervention, hourly concentrations of PM_10_, SO_2_ and polycyclic-aromatic-hydrocarbons (PAHs) were analysed.

**Results:**

PM_10_ concentrations in the intervention neighbourhood showed a peak just a few months before the introduction of the law. When compared to the control series, PM_10_ concentrations were constantly higher throughout the entire study period. After the intervention, there was a reduction in the difference between the two time-series (− 25.6%). During “wind days” results suggested no reduction in concentrations of air pollutants from 12 pm to 18 pm.

**Conclusion:**

Results of our study suggest revising the warning to the population. Furthermore, they evidence that in complex highly industrialised areas, air quality interventions cannot focus on only a single pollutant, but rather should consider the complex relationships between the different contaminants. Environmental interventions should be reviewed periodically, particularly when they have implications for social constraints. While the results of our study can be related only to the specific situation reported in the article, the methodology applied might be useful for the environmental management in industrial areas with similar features.

## Background

Addressing environmental issues is a complex process since these are produced by a large variety of factors and involve ecological, social, economic, and political dimensions, which are intrinsically correlated and interact with each other. The interests at stake, the different perspectives of the various social actors, and the intrinsic uncertainties and complexity of the systems make it particularly difficult to identify adequate technical-political solutions.

In terms of governance, an approach that focuses only on one aspect while neglecting the others is not able to sufficiently capture the variety of such complexity due to effects and feedbacks that are entirely unpredictable. This may be the case for interventions dealing with a single pollutant, neglecting the connections between it and other contaminants, or it could be a situation where the acceptability and social implications of an environmental measure are underestimated, resulting in an increased burden of distress on particular groups of people [1–4].

In an industrial area, the asymmetry between the weights of the economic interests compared to the public health aspects can determine which interests are represented in decision-making processes. As a consequence, this often leads to the implementation of partial environmental interventions, whose impacts are not always evident and relevant [5].

**Fig. 1:**
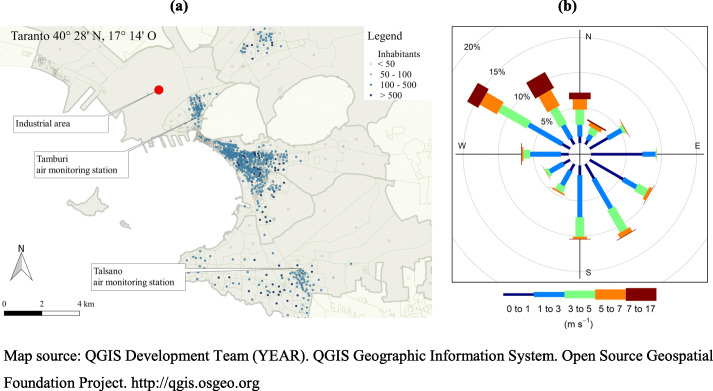
**a** The area under study, including the neighbourhood targeted by the intervention (Tamburi), the surface of the steel plant, and the monitoring stations located in Tamburi and in the neighbourhood assumed as the control (Talsano). **b** Frequency of hourly wind measurements plotted by wind direction, with colour bands showing wind speed ranges. Taranto, 2006–2016. Map source: QGIS Development Team (YEAR). QGIS Geographic Information System. Open Source Geospatial Foundation Project. http://qgis.osgeo.org

In this article, we discuss the specific case of Taranto, a southern Italian city (Fig. 1) where one of the largest steel-processing plants in Europe is situated. Several authors have reported negative health effects due to air pollution in this area [6–10]. In particular, an increased risk of mortality for all causes combined and specifically for lung cancer, respiratory diseases, and pleural mesothelioma was reported. Furthermore, excesses of cancer incidence have been observed in the youngest age classes for lymphoma and non-Hodgkin lymphoma, thyroid cancer, germ cell tumours, trophoblastic tumours, and gonad neoplasms [10].

Additionally, studies have shown associations between health outcomes and socio-economic deprivation, and identified an environmental justice issue whereas the most polluted neighbourhoods are also those with the highest deprivation [11, 12].

The steel plant in Taranto was established in 1959 as a publicly owned plant and was downsized during the 80’s and sold to a private owner in 1995. It still produced nearly 8 million tons of steel (30% of the Italian output) in 2011, respectively around 0.06 and 75% of national and provincial GDP [13]. The plant covers a surface of 15 km^2^ including 200 km of railway tracks, 50 km of roads, 190 km of conveyor belts, and large open-air mineral stockyards (Fig. 1, panel a). The wind rose in the area for the period 2006–2016 shows that the most frequent and intense winds are from the NW. Another prevailing and persistent wind system is associated with southern winds, while winds from other sectors are more due to local circulations (Fig. 1, panel b) [14].

In 2010, the Court of Taranto had requested an epidemiological and environmental study [10, 15], the results of which found that outcomes were associated with increased levels of PM_10_ originating from the industrial site, particularly among the population living close to the industrial area [15]. In the environmental study, PM emissions from the steel plant were estimated to be more than 50 tons yearly [16]. Together with other PM emissions occurring at other points of the storage cycle, the yearly amount increased to 668 tons. Storage and handling of primary materials in the stockyards are a consistent source of particulate matter generated by wind erosion. Other emissions occur at several points in the storage cycle [17]: material loading onto and out from the pile and from the movement of trucks and loading equipment into the storage pile area. After some restrictions imposed during the legal trial in 2012, the steel production was roughly halved.

In 2014, the European Commission invited Italy to urgently address this severe pollution issue arguing that: “*Italy is failing to ensure that ILVA (steel plant in Taranto) operates in conformity with EU legislation on industrial emissions, with potentially serious consequences for human health and the environment.*” In addition, “*The Commission has previously sent Italy two letters of formal notice, in September 2013 and April 2014, urging the Italian authorities to take measures in order to bring the operation of the ILVA plant into compliance with the Industrial Emissions Directive and other applicable EU environmental law*” [18].

Following the increasing pressure from citizens’ organizations and legal initiatives of the local court, air quality and public health interventions have been implemented to reduce the impact of industrial emissions. These interventions were inspired by a Canadian experience of environmental management [19].

The aim of this article is to evaluate both a law introduced by the Apulia Regional Government to mitigate the impact of industrial emissions and a warning for the population introduced by local health authorities.

The first intervention we evaluate here is the “wind-days” law [20]. This was enforced in 2012 to protect the city, especially the high-density and deprived residential area (Tamburi) located less than 1 km from the steel plant and downwind of the north/west winds (Fig. 1, panel a). This law is a compendium of norms that the steel-producing plant and other industries must adhere to in order to reduce their emissions in specific weather conditions; previously, increased concentrations of pollutants had been documented in the neighbourhood targeted by the intervention [14]. Technical details of the law are discussed elsewhere (Mangia et al. 2020). For the purposes of this paper, it suffices to know that the regional environmental authorities defined “wind days” as those during where:

i. The wind direction is in the range of 270°-360°,
ii. The speed of the wind is predicted to be greater than 6.7 m/s for at least three consecutive hours.

*Wind days* are forecasted by a meteorological modelling system 72 h in advance and communicated to the industries 48 h before windy events might occur. Following a forecasting of a wind-day, industries must implement initiatives aimed at reducing the volume and the impact of industrial activities on the neighbourhood areas.

The second intervention was enforced in 2015 and consist of a warning to the residents in Tamburi to aerate indoor environments during winter, preferably between 12 pm and 6 pm in case a wind-day is forecasted by the Regional Environmental Agency [21].

## Methods

For the years 2009–2019, PM_10_ and SO_2_ data recorded by the regional environmental authorities at the following two monitoring stations were analysed (Fig. 1):

- The first station, named “Machiavelli”, is located in Tamburi, the neighbourhood close to the industrial area and the mineral stockyards;
- The second is located in Talsano, about 13 km from the industrial area.

Daily mean PM_10_ concentrations have been computed using available hourly data. Following standard protocols, daily concentrations were discarded if more than five hourly values for one 24-h period were missing [7, 8].

In order to evaluate the “wind-day” law, average monthly PM_10_ concentrations were considered for the analysis for the period 2009–2016.

To evaluate the warning to the population, hourly concentrations of PM_10,_ PAHs (polycyclic aromatic hydrocarbons) and SO_2_ measured in the period 2015–2019 at the Machiavelli monitoring station were used to compute the pollutants’ average daily concentration profiles. Then, each day was flagged as true positive (TP), true negative (TN), false positive (FP), or false negative (FN) according to the ex-post evaluations of forecasting performed by the regional environmental authority that was made periodically available on the internet [22]. After having checked the weather conditions, we changed the classification for just 2 days (Jan 16, 2016, and May 22, 2017), updating them to FN from TN.

In order to define the impact of the “wind-days” law, an interrupted time series study design was used [23, 24].

A segmented linear regression model was implemented to study the monthly concentrations of PM_10_ in Tamburi before and after the introduction of the “wind-day” law. This design permits the evaluation of whether the intervention produced a discontinuity in comparison with the underlying secular trend [25]. In its plainest form, three coefficients of time-based covariates are estimated in the regression model, which indicates the pre-intervention slope, the change in level at the intervention time, and the change in slope from pre-intervention to post-intervention [26]. In order to minimise potential confounding, due, for example, to a change in the meteorology such as the frequency of Saharan dust incursions [27], monthly concentrations of PM_10_ in a neighbourhood 13 km away from the steel plant (Talsano) were used as a control series [28]. For power purposes, an equal number of time points before and after the intervention were assumed [29].

Thus, the following model was used [30, 31]:

$$ {Y}_t={\beta}_0+{\beta}_1{T}_t+{\beta}_2{X}_t+{\beta}_3{X}_t{T}_t+{\beta}_4Z+{\beta}_5Z{T}_t+{\beta}_6{ZX}_t+{\beta}_7Z{X}_t{T}_t+{\varepsilon}_t $$

Whereby (Fig. 2):

**Fig. 2:**
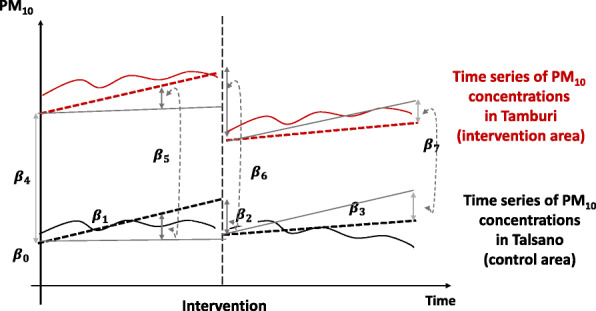
Interrupted time series design with the use of a control series

*Y*_*t*_ is the concentration of PM_10_ measured in the neighbourhood close to the industrial area (Tamburi) at each equally spaced time point *t* varying from 1 (January 2009) to 96 (December 2016).

*β*_0_ is baseline PM_10_ concentration in the control series (Talsano);

*β*_1_ is the slope of PM_10_ concentrations in the control series pre-intervention;

*β*_2_ is the change in the level of PM_10_ concentrations in the control series post-intervention;

*β*_3_ is the difference in the slopes of the PM_10_ concentrations in the control series pre-intervention and post-intervention;

*X*_*t*_ represents the intervention and is equal to 0 before the intervention and equal to 1 after;

*Z* is a dummy variable equal to 1 for the time series under study (Tamburi) and equal to zero for the control series (Talsano);

*β*_4_ is the difference in the level of PM_10_ concentrations between Tamburi and Talsano pre-intervention;

*β*_5_ is the difference in the slope of PM_10_ concentrations between Tamburi and Talsano pre-intervention;

*β*_6_ is the difference in the level of PM_10_ concentrations between Tamburi and Talsano immediately after the intervention;

*β*_7_ is the difference in the slope of PM_10_ concentrations between Tamburi and post-intervention (a difference-in-differences).

*T*_*t*_ represents the number of the months since the start of the study (1, 2, …, 96); *X*_*t*_ represents the intervention and is equal to 0 before the intervention and equal to 1 after;

*X*_*t*_ is a dummy variable. It represents the intervention and is equal to 0 before the intervention and equal to 1 after;

*Z* is a dummy variable. It is equal to 1 for the time series under study (Tamburi) and equal to zero for the control series (Talsano);

*ε*_*t*_ is the random error term.

Finally, we tested for autocorrelation using the Durbin-Watson test and adjusted regression standard errors for autocorrelation for the identified order [32]. 95% confidence intervals (95% CI) were calculated.

In order to define the impact of the warning to the population during winter, hourly concentrations of PM_10_, SO_2_ and PAHs were plotted. All days in the winter season during the period 2015–2019 were classified as follows:

- True positive days (TP), i.e. days forecasted from the regional authorities as wind-days and confirmed by later measures of meteorological variables;
- False positive (FP), i.e. days forecasted from the regional authorities as wind-days but not confirmed by later measures of meteorological variables;
- True negative days (TN), i.e. days forecasted from the regional authorities no to be wind-days and confirmed by later measures of meteorological variables;
- False negative days (FN), i.e. days forecasted from the regional authorities no to be wind-days but confirmed by later measures of meteorological variables.

## Results

**Fig. 3:**
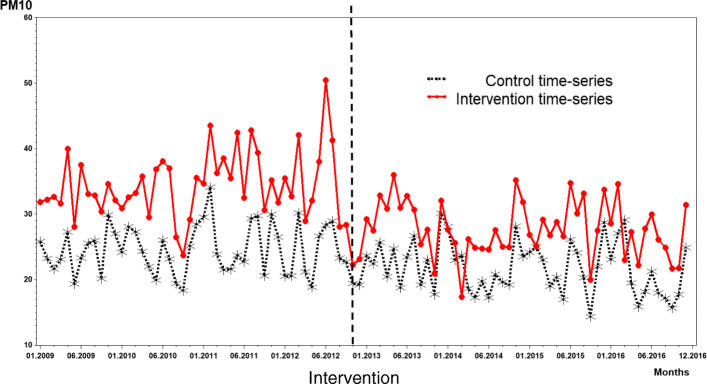
PM_10_ concentrations (μg^-2^) measured by the monitoring stations located in the neighbourhood targeted by the intervention (Tamburi) (solid red line) and in the neighbourhood assumed as the control (Talsano) (black dashed line)

From 2009 to 2016, 303 wind-days were observed. In the post-intervention period, 44% fewer wind-days were registered (*n* = 109 days). The analysis of PM_10_ concentrations in Tamburi, during the observed period showed a peak just a few months before the introduction of the law (Fig. 3). When compared to the control series (Talsano), PM_10_ concentrations were constantly higher in Tamburi throughout the whole study period. After the intervention, there was a reduction in the difference between the two time-series (Fig. 3). PM_10_ concentrations in Talsano at the beginning of the observation period were equal to 24.1 μg/m^3^ (95% CI: 22.6–25.7), while in Tamburi they were 8.2 μg/m^3^ higher (95% CI: 5.6–10.8) (Table 1). Furthermore, in the period following the intervention, the difference in level was equal to 6.1 μg/m^3^ (− 11.2 – − 1.0), i.e. 2.1 μg/m^3^ less than in the previous period (− 25.6%).

**Table 1:** Parameters estimate of the controlled interrupted time series model. Taranto. Years 2009-2016. The interruption is assumed as starting on the 1st November 2012

Parameter	Estimate	95% Confidence interval		*P*-value
β0 Baseline PM_10_ concentrations in the control series	24.1	22.6	25.7	<.0001
β1 Pre-intervention slope of PM_10_ concentrations in the control series	0.0	0.0	0.1	0.4575
β2 Post-intervention changes in level in the control series	-2.3	-4.9	0.3	0.0667
β3 Post-intervention slope in the control series	-0.1	-0.2	0.0	0.2004
β4 Baseline difference in PM_10_ concentrations level between the two time-series	8.2	5.6	10.8	<0,0001
β5 Pre-intervention difference in slopes between the two time-series	0.1	-0.1	0.2	0.1863
β6 Post-intervention difference in PM_10_ concentrations level between the two time-series	-6.1	-11.2	-1.0	0.0097
β7 Post-intervention difference in slopes between the two time-series	-0.1	-0.2	0.1	0.5768

No difference in slopes pre- and post-intervention were observed (Table 1).

From 2015 to 2019, 159 days in the winter season were forecasted as wind-days. One hundred twenty-eight of them confirmed as wind-days by later measures of meteorological variables (true positive: 80.5%) (Table 2). One thousand five hundred seventy-three days were not forecasted as wind-days. However, 1531 of them were confirmed as not being wind-days (true negative: 97.3%) (Table 2).

**Table 2:** Classification of all days in the winter season during the period 2015-2019 as forecasted and/or confirmed to be wind days or not

Confirmed by later measures of meteorological variables to be a wind-day	Forecasted as wind day				Total
	Yes		No		
Yes	128	True prositive	42	False negative	170
No	31	False positive	1,531	True negative	1,562
**Total**	**159**		**1,573**		**1,732**

**Fig. 4:**
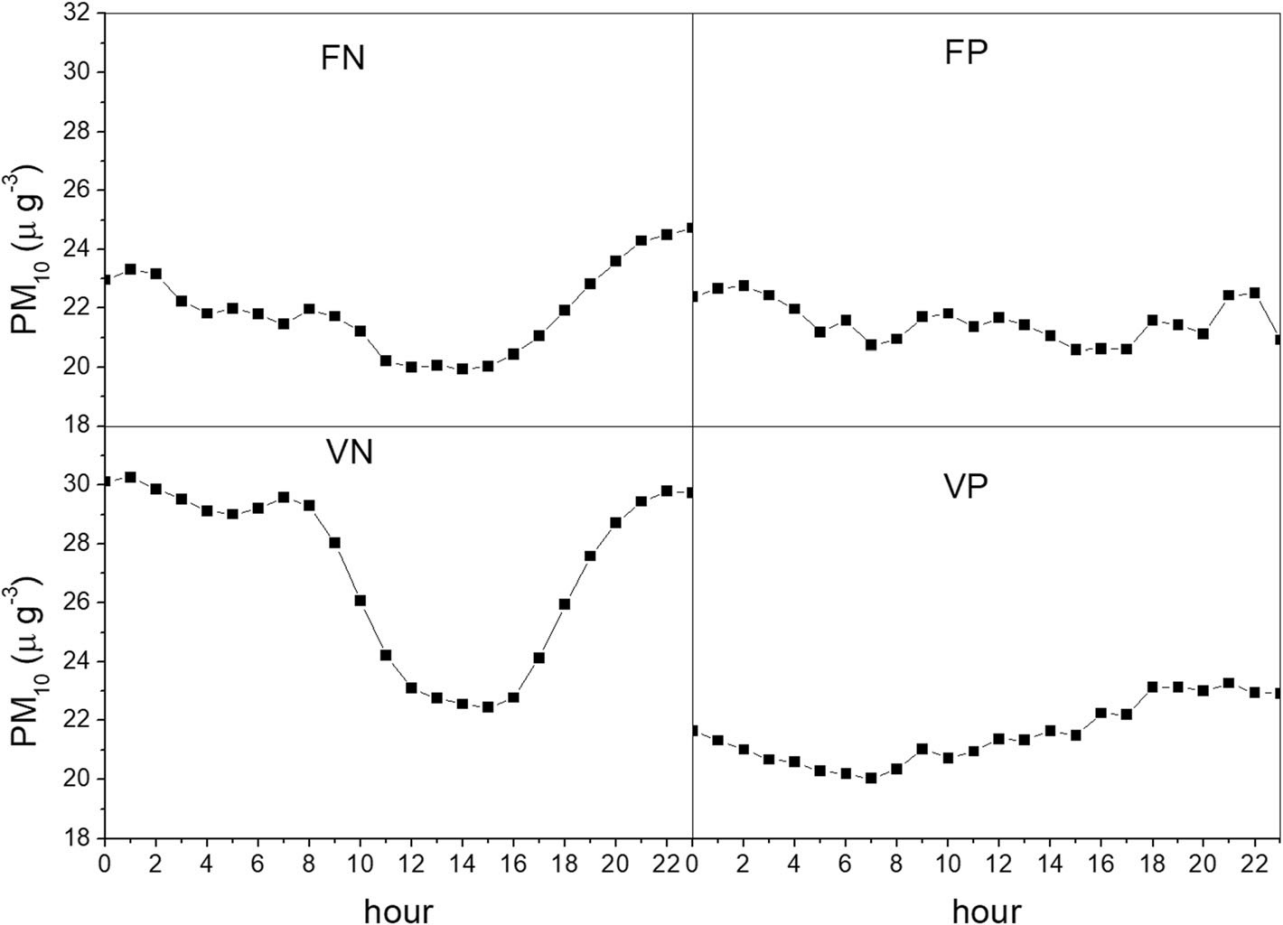
Average daily profile of PM_10_ for the years 2015–2019, from January to May and from September to December. Taranto, monitoring station Machiavelli (Tamburi)

Figure 4 shows the average daily profile of PM_10_ at the station for 2015–2019 for the months January to May and September to December, to which the warning to aerate indoor spaces from 12 pm to 6 pm, when PM_10_ concentrations are believed to be lower refers. The daily profile depends on meteorological conditions. During correctly predicted wind days (true positive), concentrations do not tend to decrease in the central hours of the day, i.e. the time period recommended to the population as ideal for indoor aeriation, but rather show an upward trend. Conversely, on true non-wind days (TN), there is a clear reduction in concentrations from 12:00 to 18:00. This different behaviour could be due to the prevalence of wind transport conditions with respect to the convective motion during highly windy conditions. The latter could inhibit the development of a boundary layer, with a consequent lower dilution of pollutants emitted from ground.

It is interesting to note that in the unforeseen wind-days (false negative) there is a reduction of concentrations in the central hours. This could be related to the fact that wind conditions are not strong enough to be predicted adequately with a meteorological model and to influence the atmospheric boundary layer [27]. During false positive days (FP), the concentration profile is more flattened. This could also be due to some effect of the measures given to the companies or particular weather conditions to be further investigated with a larger dataset (Fig. 4).

**Fig. 5:**
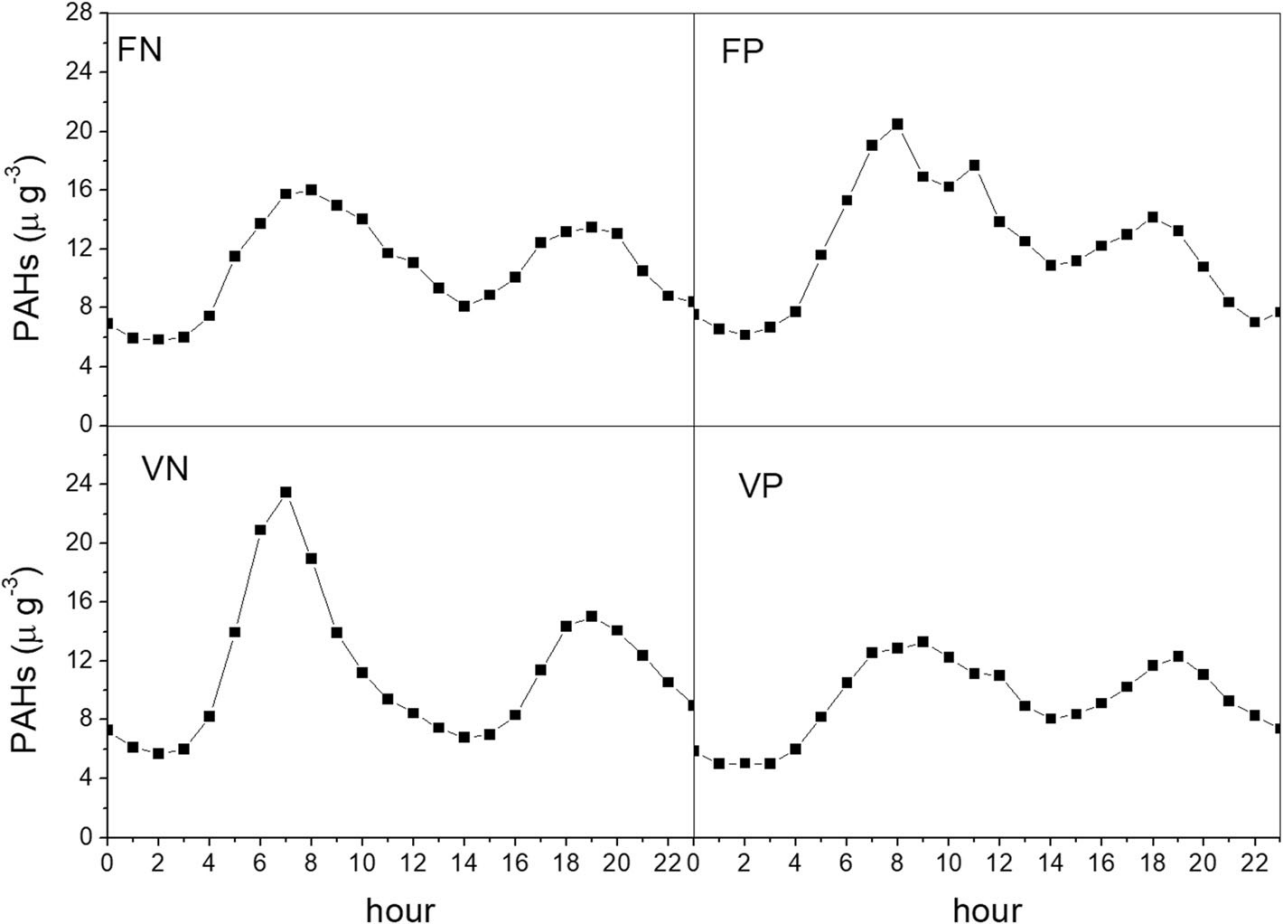
Average daily profile of PAHs for the years 2015–2019, from January to May and from September to December. Taranto, monitoring station Machiavelli (Tamburi)

The highest values of polycyclic aromatic hydrocarbons (PAHs) are recorded on non-wind days – both true negative and false positive – especially during the first half of the morning. A possible explanation is that weather conditions such as calm wind may establish a short-range diffusion of pollutants. Since the control of benzo [a] pyrene (one chemical among the PAHs) was one reason to establish rules for minimizing the impact of industrial activities, this result supports the thesis that wind days are not a unique weather condition impacting the Tamburi neighbourhood (Fig. 5).

**Fig. 6:**
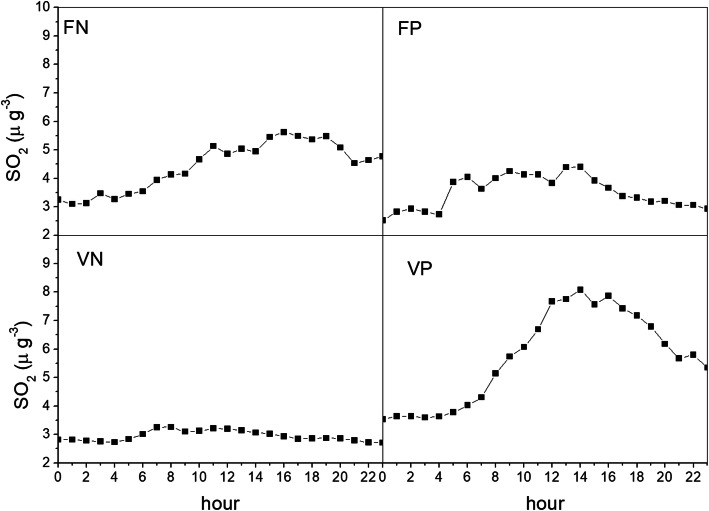
Average daily profile of SO_2_ for the years 2015–2019, from January to May and from September to December. Taranto, monitoring station Machiavelli (Tamburi)

Figure 6 shows the hourly trend of SO_2_ as a proxy of industrial combustion pollutants [14]. The profiles are very different from those observed for the PM_10_ in the four categories. During wind days, SO_2_ concentrations are higher than in all other weather conditions, with a marked increase in the central hours of the day, i.e. the time range that is the subject of the population warning.

The difference in the behaviour of the PM_10_ and SO_2_ can be explained by the different emission sources. While a large contribution to the ground concentrations of PM_10_ comes from surface sources, SO_2_ concentrations are more related to industrial combustion processes and come from sources at different altitudes (in the case of Taranto from heights of 10 to 312 m). The rise of plumes from combustion sources is strongly affected by external weather conditions. Very strong wind conditions tend to inhibit such a rise, with a consequent decrease of the plume dilution and increase of the ground concentrations.

## Discussion

In the general settings of highly industrial and highly polluted city, this study aimed to evaluate two of the most recent air-quality and public health interventions. In accordance with the original idea of improving air quality in the residential area close to the steel plant, after the introduction of the specific regional law, a strong reduction in PM_10_ concentration was observed (− 25.6%).

To evaluate the impact of the first public health intervention, an interrupted time series design was used. This study design is considered to be the most effective and powerful tool among quasi experimental designs, and an important tool for specific intervention evaluation [33]. In order to control for potential confounders, a control time-series was considered in the model. Some authors argue that the two series should not differ before the intervention i.e. they should be similar in terms of slope and level [31]. Here, this condition was met only for the slope. In fact, both series differed only in the baseline level of PM_10_ concentrations. However, this condition was planned not to be met in order to allow consideration of a neighbourhood (Talsano) 13 km away from the steel plant that is less massively affected by the industrial emissions. An assumption of the interrupted time series study design is the continuity assumption, i.e. the absence of co-interventions [34]. In our study, a considerable decrease of production might have acted as a confounding factor to the evaluation of the intervention. However, because this information was not available at a monthly level, this factor was not accounted for in the regression model. Therefore, the effect of the intervention is likely overestimated and the observed reduction in PM_10_ is partially due to the concomitant contraction of steel production. Seasonality might bias the results if not accounted for [33]. However, since we used a control series and considered an almost even distribution of winter and summer months before and after the intervention, results can be expected not to be biased [24]. The analysis of rainfall shows fluctuations in accumulated rainfall, decreasing in the post-intervention period by about 18% compared to the first period. In the interrupted time series analysis, we did not account for this potential confounder. However, rainfall is accounted for by design, while considering a control series. Thus, even if we cannot completely exclude that the amount and frequency of precipitations differ between the intervention- and the control-series, the confounding effect might be supposed to be residual.

For the second intervention, results suggested no reduction in concentrations of air pollutants from 12 to 18 pm. Thus, a revision of this warning to the population is needed.

One of the limitations of the study is the scarcity of monitoring stations and pollutant measurements in the area. The available measured pollutants, SO_2_ and PM_10_, are only partially representative of the pollutants targeted by the interventions. Specifically, there is no dimensional analysis of the dust emitted by the plant, nor is there a continuous measurement of benzo [a]pyrene. Furthermore, an analysis of PM_2.5_ concentrations was not feasible because data were not available in the period under study at the monitoring station used as control.

## Conclusions

The results indicate the need to revise the warning given to the residents during wind-days. In fact, there is no evidence supporting it since under certain circumstances an increase in pollutant concentrations has been observed from 12 pm to 18 pm. Thus asking people to aerate indoor spaces in this time window might potentially harm their health.

In conclusion, while the results of our study can be related only to the specific case reported in the article, the methodology applied might be useful for the environmental management in industrial areas with similar features.

Policy recommendations.

In the light of the current study, a number of recommendations arise:

1. In complex highly industrialised areas, air quality interventions cannot focus on only a single pollutant. One has to consider the complex relationships between the different contaminants and focus on a set of targeted pollutants.
2. If an intervention is planned to be implemented on specific days, data analysis focusing on those specific days is required. In fact, unspecific analyses can be misleading.
3. Environmental interventions should be implemented with regular planned evaluation points. In addition, scenarios with potential changes and/or adjustments have to be anticipated.
4. Environmental interventions should be reviewed periodically, particularly when they have implications for social constraints.

## Data Availability

Rough data used in our article is public and available at the following links: Meteorological data: http://www.webgis.arpa.puglia.it/meteo/index.php Data on air quality: http://old.arpa.puglia.it/web/guest/qualita_aria The datasets used and/or analysed during the current study are also available from the corresponding author on reasonable request.

## Abbreviations

EU: European Union
FN: False negative
FP: False positive
GDP: Gross Domestic Product
PAHs: Polycyclic Aromatic Hydrocarbons
PM: Particulate Matter
PM_10_: Particulate Matter with aerodynamic diameters smaller than 10 μm
SO_2_: Sulphur dioxide
TN: True negative
TP: True positive
